# Early Chk1 Phosphorylation Is Driven by Temozolomide-Induced, DNA Double Strand Break- and Mismatch Repair-Independent DNA Damage

**DOI:** 10.1371/journal.pone.0062351

**Published:** 2013-05-07

**Authors:** Motokazu Ito, Shigeo Ohba, Karin Gaensler, Sabrina M. Ronen, Joydeep Mukherjee, Russell O. Pieper

**Affiliations:** 1 Department of Neurological Surgery, University of California-San Francisco, San Francisco, California, United States of America; 2 Department of Hematology/Oncology, University of California-San Francisco, San Francisco, California, United States of America; 3 Department of Radiology and Biomedical Imaging, University of California-San Francisco, San Francisco, California, United States of America; 4 The Brain Tumor Research Center, University of California-San Francisco, San Francisco, California, United States of America; Faculdade de Medicina, Universidade de São Paulo, Brazil

## Abstract

Temozolomide (TMZ) is a DNA methylating agent used to treat brain cancer. TMZ-induced O6-methylguanine adducts, in the absence of repair by O6-methylguanine DNA methyltransferase (MGMT), mispair during DNA replication and trigger cycles of futile mismatch repair (MMR). Futile MMR in turn leads to the formation of DNA single and double strand breaks, Chk1 and Chk2 phosphorylation/activation, cell cycle arrest, and ultimately cell death. Although both pChk1 and pChk2 are considered to be biomarkers of TMZ-induced DNA damage, cell-cycle arrest, and TMZ induced cytotoxicity, we found that levels of pChk1 (ser345), its downstream target pCdc25C (ser216), and the activity of its upstream activator ATR, were elevated within 3 hours of TMZ exposure, long before the onset of TMZ-induced DNA double strand breaks, Chk2 phosphorylation/activation, and cell cycle arrest. Furthermore, TMZ-induced early phosphorylation of Chk1 was noted in glioma cells regardless of whether they were MGMT-proficient or MGMT-deficient, and regardless of their MMR status. Early Chk1 phosphorylation was not associated with TMZ-induced reactive oxygen species, but was temporally associated with TMZ-induced alkalai-labile DNA damage produced by the non-O6-methylguanine DNA adducts and which, like Chk1 phosphorylation, was transient in MGMT-proficient cells but persistent in MGMT-deficient cells. These results re-define the TMZ-induced DNA damage response, and show that Chk1 phosphorylation is driven by TMZ-induced mismatch repair-independent DNA damage independently of DNA double strand breaks, Chk2 activation, and cell cycle arrest, and as such is a suboptimal biomarker of TMZ-induced drug action.

## Introduction

Temozolomide (TMZ) is a chemotherapeutic DNA methylating agent used in the treatment of a variety of malignancies. Because of its favorable distribution, minimal side effects, and excellent penetration into the brain, TMZ is one of the most commonly used agents in the treatment of brain cancer [1], [2]. The compound, like other DNA methylating agents, has a complex mechanism of action. TMZ spontaneously decomposes under neutral or basic conditions to form the active DNA methylating agent 5-(3 methyltriazen-1-yl) imidazole-4-carboxamide, which in turn undergoes degradation to form a highly reactive methyldiazonium ion that methylates DNA at the N7 position of guanine, the N3 position of adenine, and the O6 position of guanine [3]. The N7-methyl guanine (N7G) and 3-methyl adenine (3 meA) lesions make up 70% and 25% of the total lesion burden, respectively [4]. These lesions are removed within hours in all cells by the base excision repair system. N7G is relatively unstable at physiologic conditions (half-life of 150 hrs) and either undergoes spontaneous depurination or is removed by the N-methylpurine DNA glycosylase (MPG, also known as alkyl-adenine glycosylase), [5] either of which result in abasic sites that are substrates for apurinic endonuclease [6]. Apurinic endonuclease generates single nucleotide gaps in the DNA that are ultimately filled by the concerted actions of DNA polymerase beta and the XRCC1/LIG3 DNA ligase complex [6]–[8]. 3 meA adducts are stable under physiologic conditions but also undergo excision by MPG, which similarly generates apurinic sites that are repaired by the concerted action of apurinic endonuclease, DNA polymerase beta, and DNA ligase [9]–[11]. The base excision repair system is rapid and efficient and as a result, N7G and 3 meA lesions are only cytotoxic if the repair process is defective or inhibited [12].

O6-methyl guanine (O6MG) lesions make up less than 5% of the total TMZ-induced lesion burden but are thought to account for the majority of the cytotoxic potential of TMZ [4], [13]. O6MG, unlike N7G or 3 meA, is stable and mispairs with thymine during the first round of DNA replication following adduction [14]. Resultant O6MG-thymine mispairs are recognized by the DNA mismatch repair (MMR) system which has evolved to remove the thymine paired with the O6MG, but not the O6MG itself. Because repair DNA synthesis reinserts the thymine opposite the O6MG, the O6MG lesions initiate what is considered to be a futile cycle of repair-resynthesis events [15]. This futile cycling, perhaps in connection with replication forks encountering the lesion site, leads to both DNA single strand breaks and double strand breaks (DSB) which occur roughly 2–3 cell cycles following TMZ exposure [16], [17]. The futile cycling, stalled replication forks, and resultant DNA damage in turn are associated with the phosphorylation of Chk1 and Chk2, inactivation of cdc25c, and cell cycle arrest with accumulation of cells at the G2/M boundary [18], [19]. Cell cycle arrest is believed to allow cells to potentially recover from TMZ-induced damage and avoid, or undergo preparation for, cell death by mitotic catastrophe, senescence, or apoptosis [20], [21]. The pathway that leads to TMZ-induced cell death can be circumvented by expression of O6-methylguanine DNA methyltransferase (MGMT), the DNA repair protein that removes O6MG lesions before they mispair with thymine [22], or by loss of function of the MMR system that is required to turn the mutagenic O6MG lesions into cytotoxic DSBs [23].

Because the futile MMR of TMZ-induced O6MG-thymine DNA mismatches is believed to generate the stalled replication forks and the DNA damage that activates Chk1 and Chk2, these molecules, as responders to DNA damage as well as regulators of cell cycle progression, have emerged as a key linkers of TMZ-induced DNA damage to cell cycle arrest and cell death [24], [25]. Furthermore, because phosphorylation of both Chk1 and Chk2 following clinically achievable doses of TMZ is noted only in TMZ-sensitive cells [26]–[28], Chk1 and Chk2 are considered to be biomarkers of TMZ-induced DNA damage and its downstream therapeutic consequences [29]. As noted, however, the repair of TMZ-induced non-O6MG DNA damage also generates regions of single-stranded DNA and DNA single-strand breaks that have the potential to activate Chk1. Furthermore, because published studies have only examined Chk1 phosphorylation/activation at time points following the resolution of TMZ-induced non-O6MG lesions (>12 hrs post TMZ exposure), or after super-physiologic concentrations of methylating agents [30], we considered the possibility that Chk1 activation following TMZ exposure may in fact be a consequence of non-cytotoxic, non O6MG, TMZ-induced DNA damage, and as such may be a sub-optimal biomarker of TMZ action. To address this possibility we examined Chk1 and Chk2 phosphorylation at both early and later time points following exposure to clinically achievable concentration of TMZ in isogenic GBM cells in which TMZ sensitivity was genetically or pharmacologically manipulated. We here report that levels of pChk1 were elevated within 3 hours of TMZ exposure in all cells regardless of TMZ sensitivity. This activation occurred long before the onset of TMZ-induced DNA DSB, Chk2 activation, and cell cycle arrest, and was temporally associated with TMZ-induced alkalai-labile DNA damage (ALD) produced by the non-O6-methylguanine DNA adducts. These results therefore show that Chk1 phosphorylation/activation, contrary to previous assumptions, is driven by TMZ-induced mismatch repair-independent, single-stranded DNA damage and is likely a suboptimal biomarker of TMZ-induced drug action.

## Experimental Procedures

### Cell Culture, Retroviral Infection, and Drug Treatment

U87MG human astrocytoma cells were obtained from the Brain Tumor Research Center, University of California, San Francisco. MMR-deficient HCT116 and MMR-proficient HCT116/3-6 human colorectal adenocarcinoma cells [19] were obtained from American Type Culture Collection (Rockville, Md.). G55 is a human glioblastoma cell line that has been passaged through nude mice and re-established as a stable xenograft cell line. The G55 cells, which were kindly donated by C. David James (Department of Neurological Surgery, UCSF) [31], grow comparably to U87 cells but because of their *in vivo* passaging maintain more of the characteristics of primary human GBM. All cells were grown in Dulbecco modified Eagle H-21 medium supplemented with 10% fetal calf serum at 37°C in a 5% CO2 atmosphere. Cells were plated at least 1 day before TMZ exposure. TMZ, O6-benzyguanine (BG), and N-acetylcysteine (NAC) were purchased from Sigma (St. Louis, MO) while ABT-888 was purchased from SelleckBio. All drugs were dissolved in DMSO (Sigma, St. Louis, MO). Unsynchronized cells were pretreated with DMSO, BG (20 µM for 2 hours), NAC (400 µM for 2 hours), or ABT888 (5 µM, 1 hour), and then treated with TMZ (100µM) for 3 hours in the presence of drugs. The final DMSO concentration did not exceed 0.1% (vol/vol). After TMZ treatment, cells were gently washed and incubated in fresh medium containing DMSO, BG (5 µM), NAC (400 µM), or ABT888 (5 µM), at 37°C. Cells were harvested at subconfluence at various time points. For the generation of cells over-expressing MGMT, 1×10^6^ U87 cells were seeded into six-well plates and transduced for 18 hours with a lentiviral vector encoding MGMT P140K and GFP driven by the MND promoter (kindly provided by Rustom Falahati, Division of Hematology/Oncology, Department of Medicine, UCSF) [32]. After 72 hours of culture, cells expressing GFP were isolated by fluorescent-activated cell sorting, and MGMT expression was confirmed by immunoblot analysis.

### Protein Extraction

For experiments using whole cell lysates, cells were washed with ice-cold PBS, scraped from the culture dish, and incubated in tissue lysis buffer containing 10 mmol/L Tris (pH 7.4), 150 mmol/L NaCl, 0.5% Igepal CA-630, 0.5% sodium deoxycholate, 1 mmol/L EDTA, 1 mmol/L EGTA, 1 mmol/L DTT, 10 mmol/L B-glycerophosphate, 1 mmol/L Na3VO4, 10 mmol/L NaF, 100 mg/mL phenylmethylsulfonyl fluoride, and 10 mg/mL aprotinin (all reagents were purchased from Sigma) for more than 1 hour at 4°C. Protein samples were stored at −80°C until use. The protein concentration of extracts was measured using Protein Assay reagent (Bio-Rad Laboratories, Hercules, CA).

### Immunoblot Analyses

Thirty micrograms of protein were subjected to SDS-PAGE and electroblotted onto a PVDF membrane (Millipore, Bedford, MA). The membrane was blocked in 5% nonfat skim milk (Bio-Rad Laboratories)/TBST [20 mmol/L Tris-HCl (pH 7.4), 150 mmol/L NaCl, 0.1% Tween 20] at 4°C for 1 hour and probed with antibodies against phosphorylated Chk1 (Ser345), phosphorylated Chk2 (Thr68), phosphorylated cdc25C (Ser216), Chk1, Chk2, cdc25C, MGMT, hMLH1, and β-actin (all Cell Signaling Technology) overnight at 4°C. Bound antibody was detected with horse radish peroxidase–conjugated secondary IgG (Santa Cruz Biotechnology) using enhanced chemiluminescence Western blotting detection reagents (Amersham Pharmacia Biotech, Inc., Piscataway, NJ).

### ATR Activity Assay

Cell lysates from U87 and G55+BG cells were generated 0, 1, 3, 6, 12, and 24 hours after TMZ treatment (3 hours, 100 µM) in immunoprecipitation buffer (50 mM Tris-HCl, pH 7.5, 50 mM β-glycerophosphate, 150 mM NaCl, 10% glycerol, 1% Tween 20, 1 mM NaF, 1 mM Na3 VO4, 1 mM DTT, 1 mM AEBSF, and 1× protease inhibitor cocktail Complete EDTA-free) and lysed by sonication, after which the lysates were incubated overnight at 4°C with an antibody targeting ATR or IgG then precipitated with agarose A/G beads (1 hour, 4°C). The beads were washed once with immunoprecipitation buffer, once with immunoprecipitation buffer containing 0.5 M LiCl, and three times with kinase buffer (50 mM HEPES, pH 7.5, 50 mM NaCl, 1 mM DTT, 10 mM MgCl2, 10 mM MnCl2, and 50 mM β-glycerophosphate). The beads were then incubated for 30 min at 30°C with 0.5 µg of the substrate PHAS-I (phosphorylated heat- and acid-stable protein regulated by insulin) (Sigma) in 30 µl of kinase buffer containing 30 µM unlabeled ATP. The reaction products were separated on a 15% SDS-polyacrylamide gel and subjected for immunoblotting with anti phospho-serine threonine substrate antibody (Cell Signaling) or anti-ATR (SantaCruz) goat polyclonal antibodies to assure equivalent kinase abundance.

### Single Cell Gel Electrophoresis (Comet Assay)

The DNA damage in the cells was measured using a Comet assay kit (Trevigen, Gaithersburg, MD) according to the manufacturer’s protocol. After trypsinization, cells were washed with ice-cold PBS and suspended in a 0.5% (wt/vol) solution of low temperature-melting agarose in PBS (pH 7.4) at 37°C and immediately layered onto Comet Slides (Trevigen). The agarose was allowed to set for 30 min at 4°C and the slides were incubated in a lysis solution (25 mM NaCl, 100 mM EDTA, 10 mM Tris base, 1% sodium lauryl sarcosinate, and 0.01% Triton X-100; provided by Trevigen) at 4°C for 30 min in the dark. For the alkali comet assay, the slides were immersed in alkaline unwinding solution (200 mM NaOH, 1 mM EDTA) and then subjected to electrophoresis in alkali buffer at 1 V/cm at 4°C for 30 minutes. For neutral comet assay, the lysis solution-treated slides were subjected to electrophoresis in neutral buffer at 1 V/cm at 4°C for 1 hour, and then immersed in DNA precipitation solution for 30 min at room temperature. After electrophoresis, the slides were dried, stained using SYBR Green (Trevigen), and observed by fluorescent-microscope. One hundred cells per treatment were analyzed using the computer-based image analysis system (Comet Assay, Perceptive Instruments Ltd.). The amount of DNA single strand breaks was quantitated and expressed as the “tail moment,” which combined a measurement of the length of the DNA migration and the relative DNA content.

### Measurement of Reactive Oxygen Species (ROS)

The Image-iT™ live green ROS detection system (Molecular Probes, Eugene, OR) was used to visualize ROS in live U87MG and G55 cells according to the manufacturer’s instructions. The measurement of ROS was based on the oxidation of 2,7-dichloro-dihydrofluorescein diacetate to yield an intracellular fluorescent compound. Briefly, U87MG and G55 cells seeded on 2-well chamber slides (Lab-TekTM) were washed with Hanks’solution after the treatment, and then reacted with Carboxy-H2 DCFDA (25 µmol/L) in Hanks’ solution for 30 min. Thereafter, cells were washed twice with Hanks’solution, and then photographed under a fluorescence microscope.

### Cell Cycle Studies

At various time points following TMZ exposure, cells attached to culture dishes were trypsinized and collected together with the cells floating in the media. Cells were then washed in PBS, fixed in 70% (v/v) ethanol, and stored for up to 2 weeks at −20°C. The cells were washed once with PBS followed by incubation in PBS containing 40 µg/mL propidium iodine (Sigma) and 200 µg/mL RNase A (Sigma) for 1 hour at room temperature in the dark. Stained nuclei were then analyzed using a Becton Dickinson FACScan (San Jose, CA) with 10,000 events per determination. FlowJo data analysis software (TreeStar, USA) was used to assess cell cycle distribution.

### Immunofluorescence

U87MG cells were seeded onto 2-well glass coverslips and the cells were incubated with TMZ (100 µmol/L, 3 hours) as described above. At various times after TMZ treatment, the cells were fixed and processed for immunofluorescence staining as described previously [33]. Briefly, the cells were washed in PBS, fixed with 4% paraformaldehyde in PBS for 15 minutes at room temperature, rinsed with PBS, blocked in 10% FBS in PBS containing 0.1% Triton-X for 1 hour at room temperature. The cells were stained with anti-Ser-139-phosphorylated H2A.X antibody (Millipore) at 1∶500 dilution followed by Alexa 647 conjugated secondary antibody (Molecular Probes, Eugene, OR) for 30 minutes at room temperature. Cells were washed, counterstained with 4 V,6 V diamidino-2-phenylindole (DAPI), and mounted with Prolong anti-fade reagent (Molecular Probes). Cells were then viewed on a fluorescence microscope using Axiovision Software.

### Statistical Analyses

Data are reported as mean ± standard error of at least three experiments. Differences in protein expression and tail moment measures derived using Comet assays were analyzed statistically using the paired t test. p<0.05 was considered statistically significant.

## Results

### TMZ-induced Chk1 Phosphorylation Precedes TMZ-induced DNA DSB and Chk2 Activation

**Figure 1:**
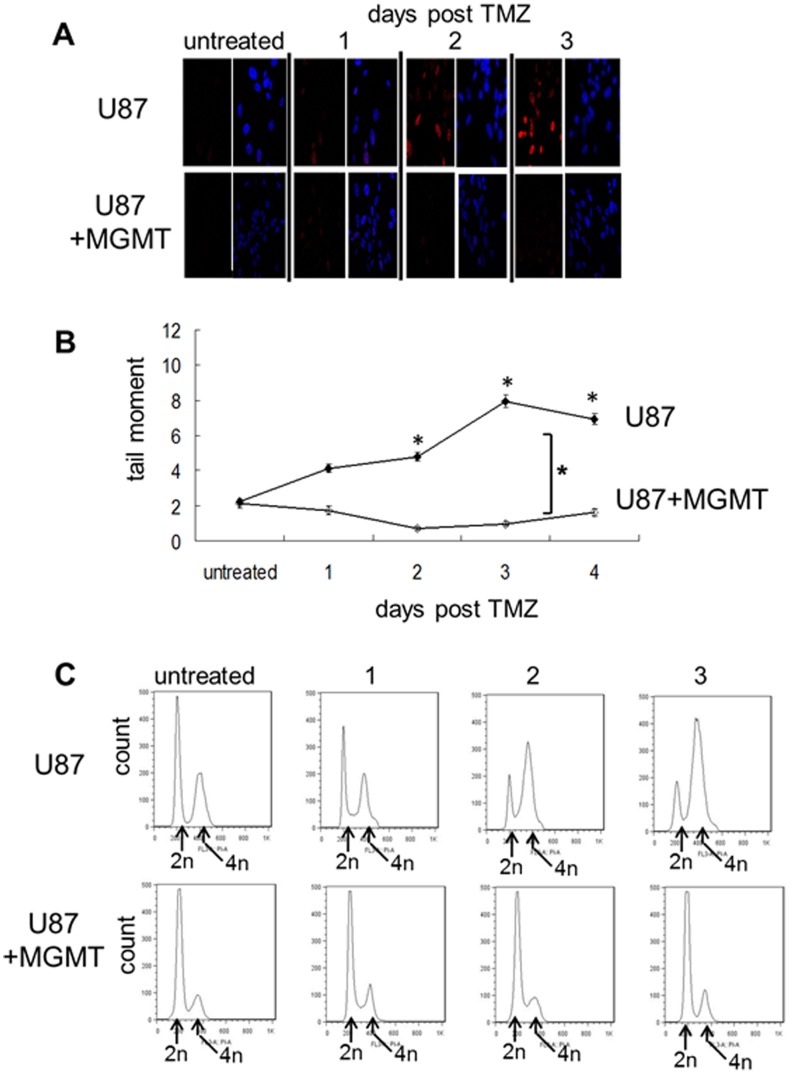
TMZ-induces delayed DNA DSB and cell cycle arrest in an MGMT-dependent manner. MGMT-deficient U87 cells or U87 cells engineered to over-express MGMT (U87+MGMT) were incubated with vehicle (untreated) or TMZ (100 µmol/L, 3 hours) after which drug was removed and cells were harvested 24, 48, and 72 hours later. A) representative photomicrographs of cells analyzed for phospho-H2A.X foci (a measure of DNA DSB) following immunofluorescent staining with anti-Ser-139-phosphorylated H2A.X antibody. Right panel of pairs, cells counterstained with DAPI to visualize nuclei. B) Analysis of tail moment (a measure of DNA fragmentation) in cells embedded in agarose, lysed, electrophoresed under neutral conditions, and stained using SYBR Green. Each point represents the mean+standard error of at least one hundred cells per treatment. *, p<.05. C) histograms of cells incubated with propidium iodide and analyzed for DNA content by FACS.

**Figure 2:**
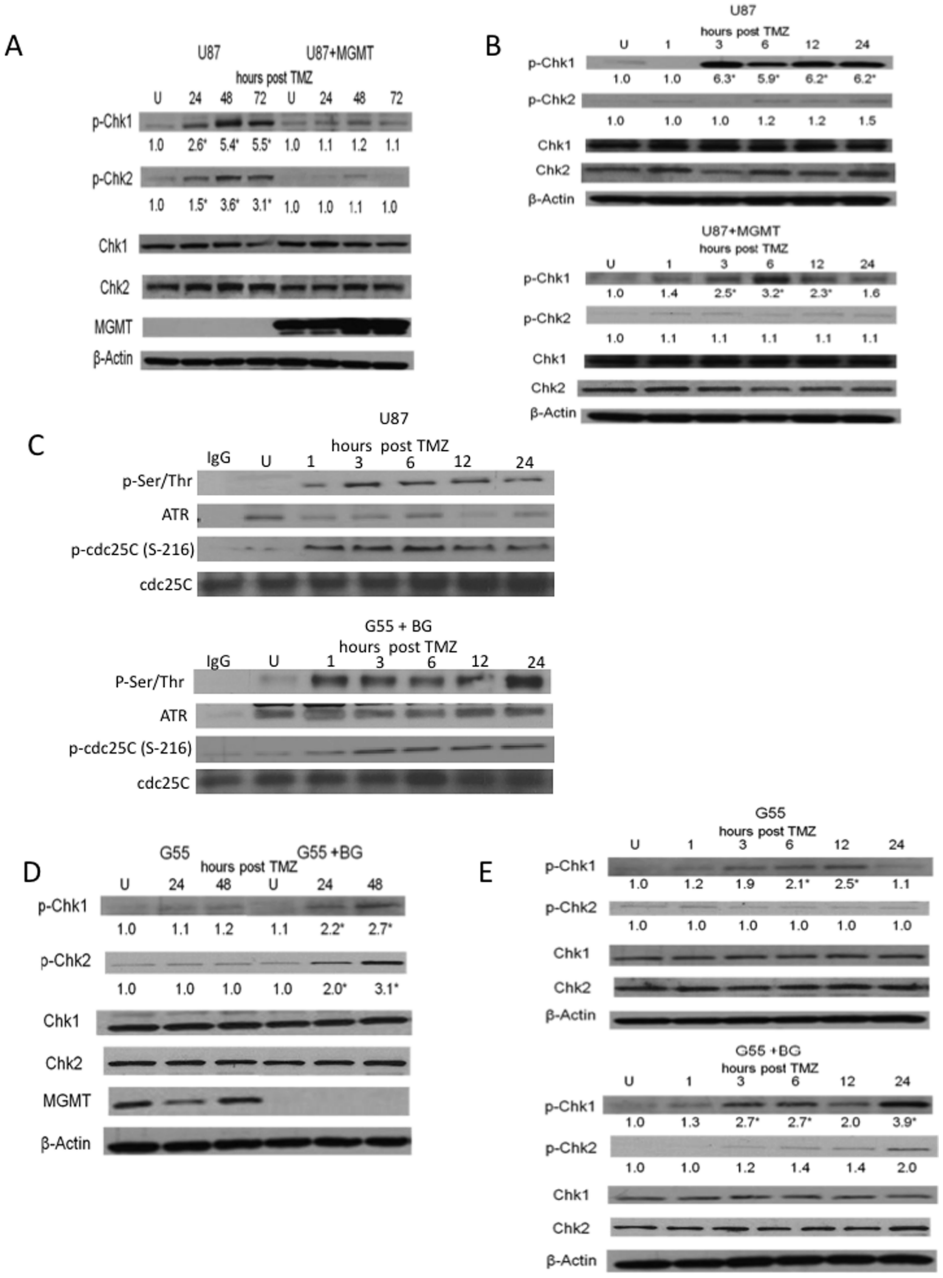
pChk1 levels are elevated prior to the onset of TMZ-induced DNA DSB, Chk2 activation, and cell cycle arrest. Isogenically paired U87 cells differing only in MGMT expression (U87 or U87+MGMT)(A- C) or G55 cells expressing MGMT or depleted of MGMT by pre-incubation with the MGMT-depleting agent BG (20 µM, 2 hours, G55+BG)(C- E) were incubated with vehicle (U) or TMZ (100 µmol/L, 3 hours) after which TMZ was removed, vehicle or BG was replaced, and cells were harvested at 24–72 hours (A, D) or at earlier time points (1–24 hours, B, C, E) following TMZ exposure for analysis of pChk1 (ser345), Chk1, pChk2 (thr68), Chk2, MGMT, pcdc25C (ser216), cdc25C, and β-actin expression by Western blot. For panel C, an ATR (or control IgG) immunoprecipitate was first prepared and incubated with the ATR substrate PHAS, after which a Western blot analysis of the extent of ATR-mediated PHAS ser/thr phosphorylation was performed. Mean fold induction of protein expression was based on densitometric measurements and is shown (relative to untreated controls) below the relevant immunoreactive bands. *, p<.05.

A 3 hour exposure to an IC50 concentration of TMZ [15] resulted in the formation of H2AX foci (a surrogate measure of DSB) beginning 2 days after drug treatment (Fig. 1A, upper panels). The fragmented DNA resulting from the DSB could also be detected in the same time frame using a single cell Comet assay performed under neutral pH conditions (Fig. 1B), as could the resulting accumulation of cells with a 4 n DNA content consistent with G2 arrest (Fig. 1C, upper panels). None of these effects were noted in isogenic U87 cells which were engineered to over-express MGMT and which repaired O6MG lesions before the O6MG lesions could mispair and cause DNA damage. Consistent with previous reports, elevated levels of pChk1 and pChk2 were also noted 2 and 3 days after TMZ exposure in MGMT-deficient U87 cells but not in U87 cells over-expressing MGMT (Fig. 2A). These results are consistent with the prevailing view that phosphorylation of Chk1and Chk2 following TMZ exposure is a biomarker of TMZ-induced DNA damage and its downstream therapeutic consequences [34].

A more detailed examination of the Chk1 phosphorylation at early times after TMZ exposure, however, revealed that pChk1 levels increased over background levels within 3 hours of TMZ exposure, and that this increase occurred in both the MGMT-deficient and MGMT-proficient U87 cells, with the induction being transient in the MGMT-proficient cells but prolonged in the MGMT-deficient cells (Fig. 2B). The phosphorylation of Chk1 in these cells paralleled the activation of ATR (as shown by the ser/thr phosphorylation of the ATR substrate PHAS by ATR immunoprecipitates of TMZ-treated U87 cells, Fig. 2C) and the Ser216 phosphorylation of the Chk1 target cdc25C (Fig. 2C), but preceded both the phosphorylation of Chk2, which was not activated for at least 24 hrs post TMZ (Fig. 2A, B), and the creation of DSB commonly thought to activate Chk2 following TMZ exposure. To examine the generality of this finding, similar studies were repeated in MGMT-proficient G55 GBM cells and isogenic cells made MGMT deficient by exposure to the selective MGMT depleting agent BG. As in the U87 cells, TMZ-induced H2AX foci, DSB, and cell cycle arrest were noted in MGMT-deficient populations (here G55+BG groups) but not in MGMT-proficient (G55) groups (not shown). Similarly, as noted in the U87 cells, elevated levels of pChk1 were noted 2 days after TMZ exposure in the MGMT-deficient G55+BG cells but not in MGMT-expressing G55 cells (Fig. 2D). However, as noted in U87 cells, pChk1 levels also increased over background levels within 3 hours of TMZ exposure in both the MGMT-deficient and MGMT-proficient G55 cells, with the induction paralleling that of ATR kinase activity and cdc25C Ser216 phosphorylation (Fig. 2C), but preceding that of pChk2, and being transient in the MGMT-proficient cells but prolonged in the MGMT-deficient cells (Fig. 2E). These results show that Chk1 phosphorylation is associated with ATR activation and cdc25C Ser216 phosphorylation but precedes TMZ-induced DNA DSB formation and Chk2 activation in two distinct GBM cell lines.

### TMZ-induced Chk1 Phosphorylation is MMR-independent

**Figure 3:**
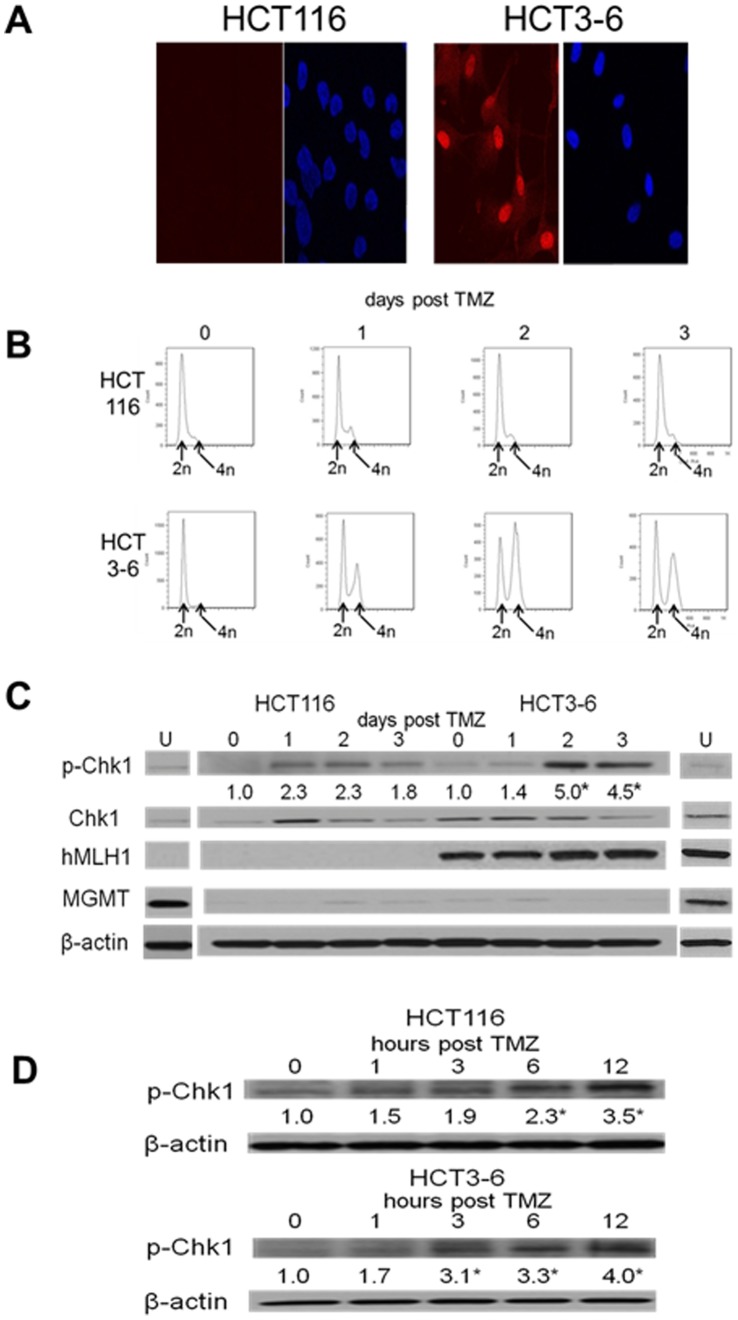
TMZ-induced Chk1 activation is MMR-independent. Isogenically paired HCT cells differing only in MMR capabilities (MMR-deficient HCT116 and MMR-proficient HCT3-6) depleted of MGMT by pre-incubation with BG (20 µM, 2 hrs, G55+BG) were incubated with vehicle (U) or TMZ (100 µmol/L, 3 hours) after which TMZ was removed, vehicle or BG was replaced, and cells were harvested at 24–72 hours (A, C) or at earlier time points (1–24 hours, B, D) following TMZ exposure for analysis of H2AX foci (A, DAPI staining in right panel of pairs), DNA content by FACS (B), or pChk1, Chk1, hMLH1, MGMT, and β-actin expression by Western blot (C, D). Mean fold induction of protein expression was based on densitometric measurements and is shown (relative to untreated controls) below the relevant immunoreactive bands. *, p<.05.

Although TMZ-induced Chk1 activation appeared to precede Chk2 activation and the formation of the DNA DSB, it remained possible that TMZ-induced, MMR-dependent single-strand breaks, or a limited number of MMR-dependent DNA DSBs induced at very early time points, were responsible for the Chk1 phosphorylation noted, at least in the MGMT-deficient cells. To address this possibility, human cells deficient in MMR by virtue of chromosome 3 loss, and the same cells complemented for MMR activity by reintroduction of chromosome 3 and gain of hMLH1 expression, were depleted of MGMT by BG exposure, then incubated with TMZ and assayed for DNA damage, cell cycle arrest, and Chk1 activation. The MGMT-deficient, MMR-deficient HCT116 cells did not exhibit H2AX foci (Fig. 3A) and did not undergo G2 arrest (Fig. 3B) at 3 days following TMZ exposure, consistent with the results of previous studies with these cells [17] and with the inability of the cells to use the MMR system to process O6MG lesions into DNA damage. In contrast, MGMT-deficient, MMR-complemented HCT3-6 cells exhibited H2AX foci and cell cycle arrest 3 days following TMZ exposure (Fig. 3A and B), consistent with the role of MMR in converting TMZ-induced O6MG lesions into DNA DSB. As with the U87 and G55 cells examined, the MGMT-deficient, MMR-proficient HCT 3–6 cells exhibited significant induction of pChk1 (but not Chk1) levels both 2 and 3 days after TMZ exposure while the MGMT-deficient, MMR-deficient paired HCT116 cells did not (Fig. 3C). An examination at early time points, however, showed that both the MMR-proficient and MMR-deficient HCT cells exhibited Chk1 activation within 3–6 hours after TMZ exposure (Fig. 3D). Because MMR-deficient cells are incapable of processing TMZ-induced O6MG lesions into DNA damage, these results show that the TMZ-induced phosphorylation of Chk1 is independent of MMR and of the DNA damage generated by this system in response to TMZ.

### TMZ-induced Chk1 Phosphorylation is Independent of the TMZ-induced Generation of ROS

**Figure 4:**
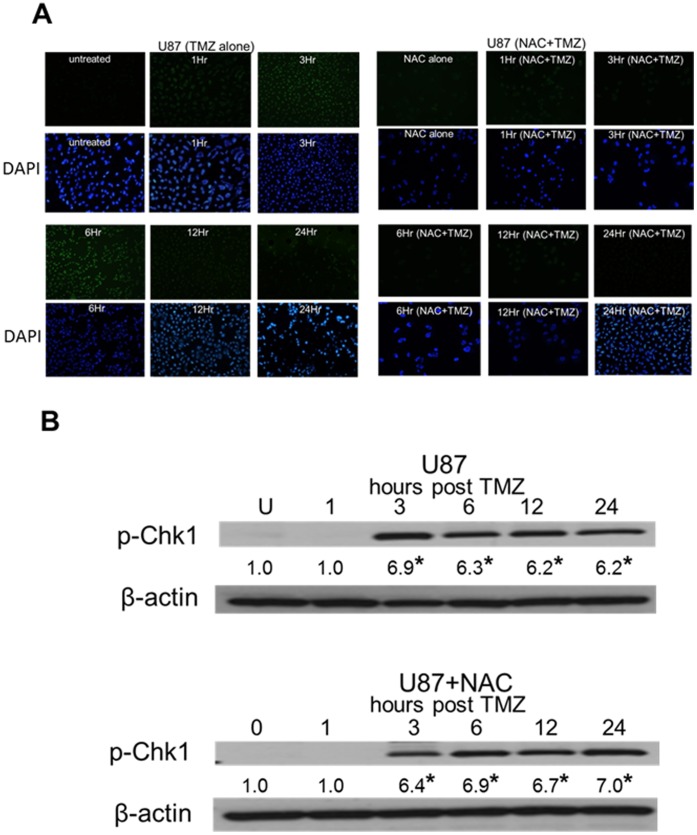
TMZ-induced Chk1 activation is independent of the TMZ-induced generation of ROS. MGMT-deficient U87 cells were pre-incubated with vehicle or the ROS scavenger NAC (400 µM, 2 hours) after which the cells were incubated with vehicle (untreated, U) or TMZ (100 µmol/L, 3 hours). TMZ was removed, vehicle or NAC was added, and the cells were harvested 1–24 hours following TMZ exposure for analysis of ROS based on the oxidation of 2,7-dichloro-dihydrofluorescein diacetate to an intracellular fluorescent compound (A, DAPI-stained cells as indicated), or pChk1 and β-actin expression by Western blot (B). Mean fold induction of protein expression was based on densitometric measurements and is shown (relative to untreated controls) below the relevant immunoreactive bands. *, p<.05.

A number of factors in addition to DNA damage have been suggested to play a role in TMZ-induced G2 arrest including most recently the generation of ROS by TMZ [35], [36]. To address the possibility that TMZ-induced ROS activate Chk1 independently of MGMT status and MMR capability, MGMT-deficient, MMR-proficient U87 cells were pre-exposed to vehicle or the reactive oxygen scavenger NAC, after which the cells were exposed to TMZ and monitored for ROS and Chk1 activation. TMZ-treated control cells began accumulating ROS within 1 hour after drug exposure (Fig. 4A, upper panel) and consistent with the data in Fig. 2B, activated Chk1 within 3 hours of TMZ exposure (Fig. 4B, upper panel). The pretreatment of the cells with NAC completely blocked the accumulation of ROS (Fig. 4A, lower panel). Elimination of the build-up of TMZ-induced ROS, however, had no effect on Chk1 phosphorylation, which occurred within 3 hours of TMZ exposure and was temporally and quantitatively indistinguishable from that noted in the TMZ-treated U87 cells in which ROS were generated (Fig. 4B, lower panel). These results show that although TMZ generates ROS in a time frame consistent with Chk1 phosphorylation, ROS do not cause Chk1 phosphorylation.

### TMZ-induced Chk1 Phosphorylation is Associated with TMZ-induced ALD

**Figure 5:**
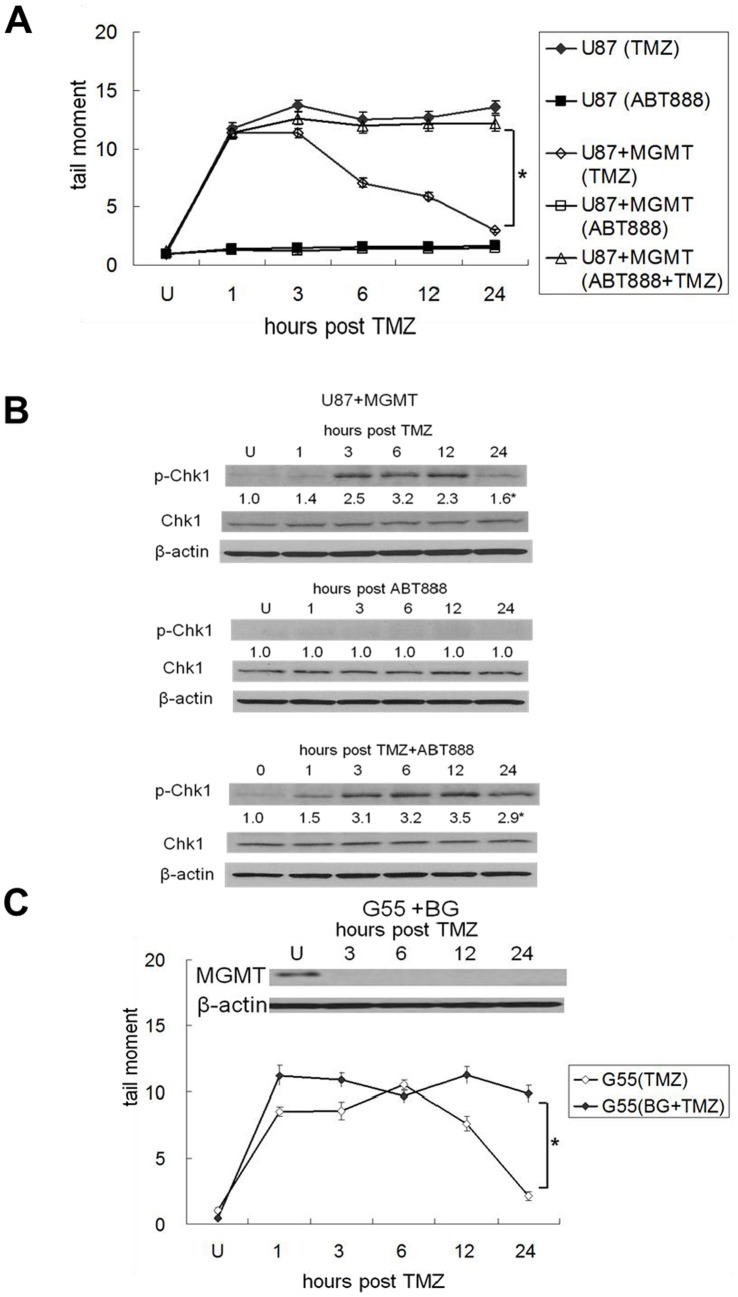
TMZ-induced Chk1 activation is associated with TMZ-induced ALD. U87 or U87+MGMT cells (A, B) were incubated with TMZ (100 µmol/L, 3 hours), ABT888 (5 µM, continuous), or both (one hr ABT pre-incubation followed by the 3 hr TMZ exposure). Cells were then washed and incubated in drug-free (for TMZ groups) or ABT-containing (for the ABT or ABT+TMZ groups ) media and harvested 1–24 hrs later for analysis of tail moment by single cell alkaline gel electrophoresis (A), or pChk1, Chk1, MGMT, and β-actin expression by Western blot (B). Tail moment values are the mean+standard error for three independent experiments. Similar tail moment and Western blot analyses were performed in G55 cells and G55 cells depleted of MGMT by a one hr pre-exposure to BG (5 µM) 1–24 hrs post TMZ (100 µmol/L, 3 hours) exposure (C). Mean fold induction of protein expression was based on densitometric measurements and is shown (relative to untreated controls) below the relevant immunoreactive bands. *, p<.05.

The observation that both MGMT-proficient and MGMT-deficient cells exhibit Chk1 activation following TMZ exposure implies that lesions unrelated to O6MG must play a role in the early activation of Chk1. In addition to O6MG, TMZ induces N7G and 3 meA DNA lesions, both of which are substrates for rapid removal by the base excision repair system but not by MGMT [37]. To address the possibility that some form of DNA damage induced by either N7G, 3 meA could underlie the TMZ-induced activation of Chk1, the paired MGMT proficient/deficient U87 and G55 cells, all of which are base excision repair- proficient [38], were exposed to TMZ, and DNA damage in the form of single strand breaks was revealed by single cell gel electrophoresis assays performed under denaturing alkaline pH conditions. The amount of ALD increased rapidly in MGMT-proficient U87+MGMT (open diamonds, Fig. 5A) and G55 cells (open diamonds, Fig. 5C) in the first hour post TMZ exposure, peaking at 3 hours and returning to control levels by 12 to 24 hours post drug. This ALD was not caused by O6MG lesions, their repair, or their downstream consequences because MGMT-mediated repair of O6MG does not involve base removal or stand breakage [38], [39], and because early induction of ALD following TMZ was also noted in cells (U87 and G55+BG) incapable of repairing O6MG (closed diamonds, Fig. 5A and C). The onset and disappearance of the ALD in MGMT-proficient cells was, however, temporally consistent with the pattern of ATR activation and Chk1 phosphorylation in these cells (Fig. 2B–D), and with the known base excision and strand cleavage processes involved in base excision repair of N7G and 3 meA lesions. Furthermore, although pre-incubation of MGMT-proficient U87+MGMT cells with the base excision repair/PARP inhibitor ABT888 alone had no effect on the extent of ALD (open squares, Fig. 5A) or Chk1 phosphorylation (Fig. 5B, middle panel), combined exposure to ABT888 plus TMZ significantly slowed the disappearance of ALD (open triangles, Fig. 5A) and prolonged TMZ-induced Chk1 activation (Fig. 5B, lower panel) relative to cells treated with TMZ or ABT888 alone. These results suggest that the early phosphorylation of Chk1 following TMZ exposure appears to be a consequence of the single-strand DNA damage (but not double-stand DNA damage) generated by the repair of TMZ-induced N7G and 3 meA lesions. In MGMT-proficient cells capable of processing TMZ-induced N7G and 3 meA lesions as well as O6MG lesions, elimination of ALD parallels the return of pChk1 to control levels. In contrast, in MGMT-deficient cells capable of processing only N7G and 3 meA lesions, unrepaired O6MG lesions and their consequences appear to lead to the sustained ALD and Chk1 phosphorylation noted up to 24 hours post-TMZ in these cells (Fig. 5A–C).

## Discussion

TMZ creates DNA adducts that lead to distinct and temporally separate forms of DNA damage. By following the formation of this damage after TMZ exposure and comparing its appearance with activation of the DNA damage response, we here show that the Chk1 phosphorylation previously linked only to TMZ-induced MMR-dependent DNA damage in TMZ-sensitive cells in fact occurs in all GBM cells examined long before the activation of Chk2, creation of MMR-dependent TMZ-induced DNA damage, and activation of the G2 checkpoint. As such these results re-define the TMZ-induced DNA damage response and show that Chk1 is likely a suboptimal biomarker of TMZ-induced drug action.

In the present studies, Chk1 was phosphorylated/activated at early time points (<12 hrs post TMZ exposure) in multiple GBM cell lines, prior to DNA DSB formation and regardless of MGMT or MMR status. These results stand in contrast to those of previous studies which, because they focused on later time points or used super-physiologic doses of methylating agents, reached very different conclusions. In studies in which cells were exposed to physiologic, near IC50 concentrations of temozolomide or the related SN1 methylating agent MNNG, and in which effects were monitored 48 hrs after drug exposure, Chk1 phosphorylation was associated with events linked to MMR-dependent processing of drug-induced O6MG lesions and drug-induced G2 arrest [26], [27]. In contrast, in studies in which concentrations of MNNG some 150-fold greater than the IC50 were used, Chk1 phosphorylation was shown to be independent of cellular MMR capacity but was associated with the creation of DNA double strand breaks, H2AX activation, and cell cycle arrest [28]. In either instance, Chk1 phosphorylation was thought to be closely associated with DNA DSB (generated either by futile MMR of O6MG-thymine mispairs or by base excision repair of high-density N-methylations), cell cycle arrest, and drug sensitivity. The results of the present study, which examined the effects of clinically achievable, IC50 concentrations of TMZ at time points prior to the formation of DNA DSB, clearly show that Chk1 phosphorylation is independent of both MMR and DNA DSB, can be separated from drug-induced cytotoxicity, and rather is driven by single-stranded forms of DNA damage resulting from non-O6MG lesions. As such these studies more clearly define the early events that occur following exposure of GBM cells to clinically achievable doses of a relevant therapeutic methylating agent.

Although our results clearly show that early Chk1 phosphorylation is a distinct entity, unrelated to MMR processing of O6MG and divorced from activation of the G2 checkpoint, the lesions that induced Chk1 activation at early time points following TMZ exposure are not well defined. In addition to O6MG, TMZ induces N7G and 3 meA lesions. These lesions do not mispair and lead to MMR-dependent DNA DSB and cytotoxicity [4], [5], [11]. 3 meA and N7G also do not lead to artifactual DNA strand breakage under the alkaline conditions used in the Comet assay, 3 meA being stable while N7G is converted to a ring-opened FAPy form that is also highly stable under alkaline conditions [37]. N7G and 3 meA do, however, both give rise to apurinic sites and DNA single-strand breaks as consequences of their repair by the base excision repair system, and are both processed in a time frame (2–12 hours) that parallels the appearance/disappearance of ALD and Chk1 activation [37]. The density of these lesions following IC50 exposures to TMZ was not sufficient to generate DNA DSB as neither H2AX foci, DNA damage detectable under neutral pH conditions, nor cell cycle arrest (not shown) were apparent in any cell type at the early time points that corresponded with early Chk1 phosphorylation. DNA single-strand breaks generated by 3 meA or N7G can, however, activate ATR as noted in the present study, and can lead to phosphorylation of Chk1 [25], [40] although if this occurs in TMZ-treated cells, it is unclear why a more immediate cell cycle arrest does not follow. One possible explanation is that full Chk1 activity requires phosphorylation of ser 345 as well as ser 317 [41]. Although TMZ-induced Chk1 ser345 phosphorylation was shown in this study to occur in the absence of a functional MMR system, other studies in cells incubated with a similar methylating agent (MNNG) showed that Chk1 ser 317 phosphorylation occurred only in MMR-proficient cells [42]. It may therefore be that the early TMZ-induced phosphorylation of Chk1 noted in the present study can occur in the absence of an activated MMR system, but is limited to ser 345, and while sufficient to lead to phosphorylation of cdc25C, is insufficient to initiate cell cycle arrest. Nonetheless, the lesions that induce Chk1 phosphorylation at early time points following TMZ exposure may be either AP sites generated by spontaneous depurination of N7G (and which appear as ALD following cleavage under alkaline assay conditions), frank DNA single-strand breaks generated following apurinic endonuclease-mediated cleavage of 3 meA- or N7G-associated AP sites, or a combination of the two. While all of these lesions are associated with the creation of single strand DNA and single-strand breaks that can cause Chk1 activation, the present study is the first to show that ser345 pChk1 activation is related to these non-O6MG TMZ-induced lesions and the subsequent single-stranded DNA damage they cause, is not unique to TMZ-sensitive cells, and as such is not likely a useful biomarker of response.

Although non-O6MG lesions appear to initiate the DNA damage response following TMZ exposure, unrepaired O6MG lesions appear to be responsible for the persistent Chk1 phosphorylation. In support of this idea, pChk1 levels returned to control values within 12 hours of TMZ exposure in base excision repair/MGMT-proficient cells, but remained elevated for at least 24 hours in those cells lacking MGMT. While it is possible that O6MG lesions were acted on by the same base excision repair system that repairs N7G and 3 meA lesions and activates Chk1, O6MG does not undergo spontaneous or alkali-induced depurination, and is a poor substrate for apurinic endonuclease or MPG-mediated removal [11], [33]. It rather appears that these O6MG lesions are processed by an alternative mechanism into at least alkali-cleavable AP sites and perhaps into unresolved DNA single-strand break intermediates. Furthermore, this system appears to be relatively efficient at converting unrepaired O6MG into ALD but relatively inefficient at fully resolving the lesions as the associated ALD persists at least 24 hours after TMZ exposure created the O6MG lesions. Thymine or guanine-thymine specific thymine glycosylases may act on unrepaired O6MG lesions, perhaps slowly or unsuccessfully, leading to prolonged Chk1 activation in MGMT-deficient cells [43]. Conversely, the late Chk1 activation may simply be a result of incomplete processing of O6MG-induced mismatches by the MMR system. These results suggest that while Chk1 is initially activated in response to N7G/3 meA lesions, mechanisms that recognize and process unrepaired and unmispaired O6MG can prolong Chk1 activation.

The present studies, in addition to more clearly defining the DNA damage response to TMZ and the role of Chk1 in the process, also have implications for the use of TMZ and the monitoring of its action. Because Chk1 phosphorylation/activation has previously been associated with TMZ-induced DNA DSB damage and cell cycle arrest, both of which are precursors to TMZ-induced cell death, Chk1 activation has been suggested to be a biomarker of TMZ activity [26]. The results of the present study, however, show that while ser345 pChk1 may be a marker of TMZ delivery to the cell and of TMZ-induced DNA damage, it is not per se an indicator of therapeutic response. Nonetheless the early Chk1 phosphorylation following physiologic exposures to TMZ is of biologic consequence as the monitoring of the timing of Chk1 activation could potentially provide information as to the functionality of repair systems in the cell, with early and transient ser345 Chk1 phosphorylation being a marker of functional base excision repair, and prolonged ser345 Chk1 phosphorylation being a marker of lack of MGMT. Additionally, the differences in Chk1 phosphorylation noted between the TMZ-sensitive and TMZ-resistant cells used in this study have recently been shown to be related to changes in pyruvate metabolism detectable by non-invasive magnetic spectroscopy imaging [44]. In this way knowledge of the sequence of events that occur following exposure to therapeutically relevant concentrations of TMZ may not only improve our understanding of the links between the DNA damage response and the control of cellular metabolism, but also contribute to the development of methods that allow real-time monitoring of TMZ response, and improved care for individuals with brain tumors.
